# Salmonella enterica Serovar Typhi on an Island: No H58, No Multidrug Resistance, but for How Long?

**DOI:** 10.1128/mbio.02426-22

**Published:** 2022-12-05

**Authors:** Arif Mohammad Tanmoy

**Affiliations:** a Child Health Research Foundation, Dhaka, Bangladesh

**Keywords:** typhoid fever, antimicrobial resistance, H58, typhoid conjugate vaccine, multidrug resistance

## Abstract

Little genomic data is available for typhoid fever from island nations, though the disease has a moderately high burden there. Sikorski et al. (M. J. Sikorski, T. H. Hazen, S. N. Desai, S. Nimarota-Brown, et al., mBio 13:e01920-22, 2022, https://doi.org/10.1128/mbio.01920-22) studied 306 Salmonella enterica serovar Typhi genomes from the Samoan Islands collected during 1983 to 2020 and reported dominance of a rare genotype, 3.5.4, and no H58 (genotype 4.3.1). They found pansusceptibility of all isolates to three first lines of antimicrobial agents (ampicillin, chloramphenicol, and cotrimoxazole). This commentary evaluates the importance of these findings for the Samoan Islands and how they can help the global typhoid community. The microbial community in the environment and human gut could have played a role in the lack of antimicrobial resistance (AMR). However, drug-resistant strains may arrive soon at the island, as their international spread is common. Further investigation would help the global typhoid community to better understand the evolution of an isolated pathogen community and the effect of vaccination there.

## COMMENTARY

The genomics of Salmonella enterica serovar Typhi, the causative agent for typhoid fever, has come a long way in the last 2 decades. The first complete Salmonella Typhi genome was published in 2001, that of a multidrug-resistant strain named CT18 (1). Since then, genomic data of almost 8,000 isolates have been published; the majority of these isolates have been derived from countries with a high burden of typhoid fever (2). These data have played an instrumental role in understanding the evolutionary diversity of the pathogen, as well as the spread of multidrug-resistant genotype 4.3.1, or H58, which disseminated from South Asia to almost every part of the world (2, 3). During the last decade, another highly resistant lineage of Salmonella Typhi, with extensively drug-resistant (XDR) characteristics, originated in South Asia and started an outbreak in Pakistan in 2016 (4). It has now spread to at least 14 different countries, including countries where typhoid is not endemic. However, unlike the high-burden regions like South and Southeast Asia or high-income countries like the United Kingdom, availability of genomic data from regions with moderately high typhoid fever burden is still rare. One such region is Oceania, which consists of multiple small island nations (except for Australia and New Zealand). To date, only two studies have been reported from this region (from Papua New Guinea and Fiji) with genomic data analyses of Salmonella Typhi isolates (5, 6). Therefore, a lack of proper understanding exists regarding the emergence, population diversity, and antimicrobial resistance (AMR) of Salmonella Typhi there.

A report by Sikorski et al. (7) in a recent issue of *mBio* looked into a genome data set from the Samoan Islands, one of those nations. The authors sequenced 201 Salmonella Typhi isolates and added 105 previously published historical genomes from the island to build a database of 306 isolates, collected over 38 years from 1983 to 2020. Their analysis portrayed the population structure and genotypic diversity of Salmonella Typhi in the Samoan Islands. Remarkably, they found dominance of genotype 3.5.4 (93%; 285/306), which is rare outside the islands according to previous reports (3). The genotype seems to be uniquely preserved on the islands and showed three different sublineages, 3.5.4.1, 3.5.4.2, and 3.5.4.3, all of which have been defined by Sikorski et al. using single nucleotide polymorphisms (SNPs) (7). Considering that most of the non-Samoan typhoid cases of genotype 3.5.4 can be linked to cases who travelled from or via that island, the genotype can be considered as exclusively local to the Samoan Islands. The authors reported that the most recent common ancestor (MRCA) of genotype 3.5.4 was estimated to exist during the early 1970s (7).

Interestingly, no multidrug-resistant (MDR) isolate of Salmonella Typhi was found in the islands. No isolate resistant to any of the first-line antimicrobial agents for typhoid treatment, penicillin (ampicillin or amoxicillin), cotrimoxazole, and chloramphenicol, was found either. Indeed, these drugs are still in the market of the island and are being used for typhoid treatment (8, 9). No H58 or genotype 4.3.1 isolates were reported either (2). There are reports of over-the-counter sales of antibiotics in Samoa, which raises the question of why the Salmonella Typhi population there has not acquired high rates of AMR yet. A major cause of that could be the difference in the environmental microbial community in the island. The presence of resistance genes in other pathogens from the island, including Escherichia coli and Klebsiella spp., has been reported, and the genes are usually located on R-factor plasmids (9). In contrast, the AMR genes in Salmonella Typhi are usually carried by an IncHI1 plasmid, especially the ones that are responsible for MDR. Laboratory experiments suggest that an R-factor plasmid transferred into a Salmonella Typhi cell cannot be stably maintained (10). This indicates a possibility that Salmonella Typhi in Samoa has not acquired the MDR genes due to incompatibility with the plasmid types available in its environmental niche. Thus, the most efficient way for the Salmonella Typhi community in Samoa to acquire AMR genes is from bacteria brought in by travelers. Considering that the island is a popular tourist destination, importation of highly resistant Salmonella Typhi genotypes like 4.3.1 with MDR genes or mutations in the quinolone resistance-determining regions (QRDRs) is an imminent threat, and if this occurs, it could replace the local Salmonella Typhi population.

The dominance of genotype 3.5.4 in the Samoan Islands could be related to the diversity of the host gut microbiome as well. Although the resistance genes can be transferred horizontally within the microbial community of the gut (11), the same “R-factor plasmid versus IncHI1” incompatibility could hinder the Salmonella Typhi community from acquisition of the genes. However, dominance of one Salmonella Typhi lineage in an isolated island nation is not unique to Samoa. It has also been reported from Papua New Guinea with genotype 2.1.7. The same report observed chromosomal rearrangements near the *rrn* operon that can be associated with host persistence and carriage through the gallbladder (5, 12). No such changes were reported by Sikorski et al. for Samoan genomes. They found high conservation of a 106-kb long pHCM2 plasmid, though (7). The presence of this cryptic plasmid in Samoa was high in comparison to global data (35% versus 5%), highlighting its stability within the Samoan Salmonella Typhi population. However, the plasmid is historically known to carry genes related to DNA metabolism and replication (13), and it is not clear how it can be linked to the unique conservation of the Salmonella Typhi population in Samoa.

Continuation of the laboratory surveillance network established by the Ministry of Health of the Samoan Islands during the study by Sikorski et al. for genomic surveillance to track changes in the Salmonella Typhi population over time is highly recommended. This is vital considering the recent inauguration of a mass vaccination program for typhoid conjugate vaccine (TCV) in the Samoan Islands in August 2021 (14). Knowledge of changes in the Salmonella Typhi population of Samoa during the post-TCV period will be crucial, as there are previous reports that the typhoid vaccines can impact isolated island nations differently (15). The Samoan government has also emphasized typhoid control and declared typhoid fever a notifiable disease. In the case of landfall of travel-related drug-resistant Salmonella Typhi, the establishment of genomic surveillance for typhoid fever by the Samoan Government will likely identify such events rapidly and allow countermeasures to contain the spread of imported clones to be instituted.

The data presented by Sikorski et al. on the Samoan Islands and the high conservation of the one-genotype-dominated Salmonella Typhi population will help the global typhoid community to understand the evolution of this pathogen better, especially in an isolated community. Further investigation to understand the unique conservation and lack of AMR in the Salmonella Typhi population and their changes with vaccines would help the typhoid community gain knowledge on how an intervention can affect isolated Salmonella Typhi populations and how this experience can be useful to control the typhoid burdens in other countries.
